# Ingestion of High β-Glucan Barley Flour Enhances the Intestinal Immune System of Diet-Induced Obese Mice by Prebiotic Effects

**DOI:** 10.3390/nu13030907

**Published:** 2021-03-11

**Authors:** Kento Mio, Nami Otake, Satoko Nakashima, Tsubasa Matsuoka, Seiichiro Aoe

**Affiliations:** 1 Studies in Human Life Sciences, Graduate School of Studies in Human Culture, Otsuma Women’s University, Chiyoda-ku, Tokyo 102-8357, Japan; mio.kento@hakubaku.co.jp (K.M.); otake.nami@gmail.com (N.O.); 2 Research and Development Department, Hakubaku Co. Ltd., Chuo-City, Yamanashi 409-3843, Japan; nakashima.satoko@hakubaku.co.jp (S.N.); matsuoka.tsubasa@hakubaku.co.jp (T.M.)

**Keywords:** barley, β-glucan, short-chain fatty acids, immune system, sIgA, prebiotics, microbiota

## Abstract

The prebiotic effect of high β-glucan barley (HGB) flour on the innate immune system of high-fat model mice was investigated. C57BL/6J male mice were fed a high-fat diet supplemented with HGB flour for 90 days. Secretory immunoglobulin A (sIgA) in the cecum and serum were analyzed by enzyme-linked immunosorbent assays (ELISA). Real-time PCR was used to determine mRNA expression levels of pro- and anti-inflammatory cytokines such as interleukin (IL)-10 and IL-6 in the ileum as well as the composition of the microbiota in the cecum. Concentrations of short-chain fatty acids (SCFAs) and organic acids were analyzed by GC/MS. Concentrations of sIgA in the cecum and serum were increased in the HGB group compared to the control. Gene expression levels of *IL-10* and polymeric immunoglobulin receptor (*pIgR*) significantly increased in the HGB group. HGB intake increased the bacterial count of microbiota, such as *Bifidobacterium* and *Lactobacillus*. Concentrations of propionate and lactate in the cecum were increased in the HGB group, and a positive correlation was found between these organic acids and the *IL-10* expression level. Our findings showed that HGB flour enhanced immune function such as IgA secretion and *IL-10* expression, even when the immune system was deteriorated by a high-fat diet. Moreover, we found that HGB flour modulated the gut microbiota, which increased the concentration of SCFAs, thereby stimulating the immune system.

## 1. Introduction

The gastrointestinal tract has to tolerate the presence of the luminal microbiota, but the immune system must protect the intestinal mucosa against potentially harmful dietary antigens and pathogenic agents [1]. Immunoglobulin A (IgA) is an important antibody of this system, which eliminates pathogens and neutralizes toxins. It is known that this system is aggravated by stress, obesity, and disordered eating habits [2]. In particular, foods and food ingredients have the potential to affect the intestinal immune system. Previous studies showed that several food components, such as lactic acid bacteria and vitamin A, promote IgA secretion by different mechanisms [3,4,5]. Additionally, it is reported that functional foods stimulate IgA secretion by inducing changes in the gut microbiota [6,7]. Indeed, homeostasis of gut microbiota has been shown to be regulated by T cell-dependent IgA [8]. Moreover, a human study indicated that IgA-deficient subjects have different microbiota profiles compared with healthy subjects [9]. Therefore, it is important to identify foods that enhance intestinal immune functions, such as IgA production, by improving gut microbiota.

In recent years, there is increasing interest in utilizing indigestible carbohydrates, to modulate the metabolic function of the microbiota, which are known as prebiotics [10,11]. Grains such as barley contain substantial amounts of dietary fiber and mediate several physiological functions. Beta-glucan in barley and oats is a water-soluble dietary fiber comprising β-glycosidic bonds (β-1-3,1-4 bonds). This fiber is fermented by gut bacteria, potentially leading to health benefits, and thereby acting as a prebiotic [12,13]. A previous study showed that a mixture of barley β-glucan and probiotic microorganisms modulate the transcriptional levels of immune-related genes in vitro [14]. Additionally, whole-grain barley pasta containing barley β-glucan was found to be effective in modulating the composition and metabolism of the gut microbiota in 26 healthy subjects [15]. Another clinical study showed that intake of 60 g/day of whole barley for four weeks increased microbial diversity and the abundance of many genera of gut bacteria [16]. Moreover, β-glucan from oats and barley contribute to the production of intestinal metabolites, such as short-chain fatty acids (SCFAs), via fermentation mediated by gut microbiota [17]. SCFAs are thought to stimulate the immune system and orchestrate an anti-inflammatory effect [18,19]. Park et al. suggested that SCFAs, such as butyrate, propionate, and acetate, activate the naive T cell polarization to regulatory T cells (Tregs) [20]. Another in vitro study showed that butyrate suppressed the induction of T cell apoptosis and interferon gamma (IFN-γ)-mediated inflammation in colonic epithelial cells [21]. Therefore, changes in the gut microbiota and levels of SCFAs following intake of barley are expected to enhance the immune system in the lower gut.

We speculated that barley β-glucan may have an effect on the innate immune function via intestinal fermentation. To date, few studies have focused on changes to the immune system following barley intake. One such study involved patients who had previously undergone a proctocolectomy with ileostomy [22]. The subjects were fed oat β-glucan, and then, digestive waste was collected, freeze-dried, and dissolved in PBS. The resulting solution was incubated with different intestinal cell lines and found to increase parameters related to immune modulation in vitro. Volman et al. suggested that mice fed oat β-glucan activated the gut leukocytes and enterocytes compared to placebo mice [23]. However, most of the studies were conducted using β-glucan extracts, and there have been no studies evaluating barley as a food. Furthermore, there are no reports that have clarified the effects of barley on models of impaired immune function due to poor dietary habits such as obesity. Diet-induced obesity generally causes a low-grade inflammatory state. Several lines of evidence indicate that altered immune function is associated with the etiology of obesity [24,25,26]. For example, intake of a high-fat diet causes excess lipids and secreted bile acids to flow into the digestive tract, which adversely affects the intestinal environment and immune function. A recent study showed that serum IgA levels were decreased in mice fed a high-fat diet, which was mediated via high-fat-induced changes to the composition of microbiota and gut metabolite production [27]. Therefore, it is important to investigate the effect of food containing prebiotics, such as barley, on inflammation in the intestinal tract caused by obesity.

Here, we focused on the gut fermentability of barley flour and confirmed its effect on the innate immune response under high-fat conditions. Firstly, we investigated gene expression to clarify whether the intake of barley affects the immune system in the ileum using previously published DNA microarray data [28]. Next, as the main aim of this study, we investigated whether or not IgA secretion and mRNA expression levels of cytokines change following the ingestion of high β-glucan barley (HGB) flour in diet-induced obese mice. We also investigated the relationship between gut microbiota and the production of SCFAs.

## 2. Materials and Methods

### 2.1. Animals and Study Design

The animal protocol used in this study was approved by the Otsuma Women’s University Animal Research Committee (Tokyo, Japan, No.19013, 13 December 2019) and implemented in accordance with animal experimentation according to their regulations. The flowchart of the study design in this animal experiment is shown in Figure S1. Male four-week-old C57BL/6J mice were purchased from Charles River Laboratories Japan, Inc. (Yokohama, Japan). Each mouse was individually housed in a polycarbonate cage kept in a holding room maintained on a 12 h light/dark cycle (light on at 07:30 h) at a temperature of 22 ± 1 °C and humidity of 50 ± 5%. After the mice had acclimatized for 11 days on commercial chow (NMF, Oriental Yeast Co., Ltd., Shiga, Japan), they were randomized into 2 groups according to body weight (*n* = 8 per group). Mice were given the experimental diet (powdered diet) over a 90-day period. The experimental diet was a 50% fat energy diet supplemented with cellulose (Control (C) group) or flour of waxy hulled barley “White Fiber” (high β-glucan barley (HGB) group). The total dietary fiber of each diet was adjusted to 5% (Table S1). “White Fiber” refers to barley flour rich in β-glucan (Table S1). “White Fiber” flour which was pearled to 70% and powdered was obtained from the Hakubaku Co. Ltd. (Yamanashi, Japan). Food intake and body weights were monitored 2 or 3 times per week throughout the study period. Feces were collected for 5 days in the 11th week. At the end of the study, mice were fasted for 8 h and sacrificed by isoflurane/CO_2_ anesthesia. Then, the cecum with digesta, adipose tissues (epididymal, retroperitoneal, mesenteric fats), and liver were dissected and weighed. Blood samples were collected from the postcaval vein and centrifuged to obtain serum, which was stored at −30 °C until enzyme-linked immunosorbent assay. Cecum with digesta was stored at −30 °C until analysis of SCFAs, microbiota, and secretory immunoglobulin A (sIgA). Ileum tissue was soaked in RNA^®^ protect Tissue Reagent (Qiagen, Hilden, Germany) and stored at −30 °C until extraction of total RNA.

### 2.2. Gene Expression Analysis of the Immune System Using DNA Microarray Data

The microarray data used in this study have been registered at the National Center for Biotechnology Information (NCBI) for Gene Expression Omnibus (GEO) and GEO series with accession number GSE157828. Male mice were fed a high-fat diet (fat energy ratio of 50%) supplemented with β-glucan rich barley flour before extracting total RNA from the ileum, liver, and adipose tissue and performing DNA microarray analysis. The procedure was also performed for mice fed a diet not supplemented with β-glucan rich barley flour as a control (supplemented with cellulose). We identified differentially expressed genes (DEGs) using cut-off criteria (Log-ratio > 1.3 fold and < 0.77 fold in the barley group compared with the control group) based on our previous study [28]. Kyoto Encyclopedia of Genes and Genomes (KEGG) enrichment analyses of DEGs were performed using the DAVID database (accessed on 12 December 2020). False discovery rate (FDR) was calculated using the Benjamini–Hochberg algorithm. Pathways were extracted with FDR where *p* < 0.05.

### 2.3. The Levels of Secretory Immunoglobulin A, IL-6, and IL-10 Determined by Enzyme-Linked Immunosorbent Assays

The levels of sIgA in the cecum and serum were determined using an enzyme-linked immunosorbent assay kit for secretory immunoglobulin A (Cloud-Clone Corp., Katy, TX, USA). Serum was analyzed according to the manufacturer’s instructions. Cecum was lyophilized, and 10 mg samples were added to 100 μL phosphate buffered saline (PBS) (10 mM, pH 7.0) and then homogenized. After centrifugation (8000 rpm × 20 min, 4 °C), the supernatant was diluted 500 times in PBS and then used for measurement. The concentrations of interleukin (IL)-6 and IL-10 in serum were analyzed using Mouse IL-6 ELISA Kit (RayBiotech, Inc., Norcross, GA, USA) and Mouse IL-10 ELISA Kit (Proteintech Group, Inc., Chicago, IL, USA), respectively.

### 2.4. Analysis of Short-Chain Fatty Acids in the Cecum and Feces

The concentration of cecum and feces SCFAs was determined as described in a previous report using gas chromatography-mass spectrometry (GC/MS) [29]. First, 10–20 mg of cecum digesta was added to 100 μL of internal standard (100 μM crotonic acid), 300 μL of diethyl ether, and 50 μL of HCl, and then homogenized (Tissue Lyser II; Qiagen) twice at 2000× *g* rpm for 2 min each. After centrifugation (3000 rpm, at 24 °C, for 10 min), 80 μL of supernatant was added to 16 μL of *N*-*tert*-butyldimethylsilyl-*N*-methyltrifluoroacetamide in a vial, and derivatization was performed at 80 °C for 20 min. Samples were stored at room temperature for 48 h and then analyzed for SCFAs by GC/MS (7890B GC system equipped with a 5977A MSD; Agilent, Tokyo, Japan). A DB-5MS capillary column (30 m × 0.53 mm) (Agilent) was used to separate the SCFAs. The oven temperature was initially kept at 60 °C and then ramped up to 120 °C at a rate of 5 °C/min. Then, the oven was ramped up to 300 °C at a rate of 20 °C/min and finally maintained at 300 °C for 2 min. Helium was used as the carrier gas at a flow rate of 1.2 mL/min. The temperature of the front inlet, transfer line, and electron impact ion source were set at 250, 260, and 230 °C, respectively. Mass spectral data were collected in selective ion monitoring mode. The concentration of SCFAs was calculated by comparing their peak areas with that of the internal standard.

### 2.5. Analysis of Counts of Predominant Bacterial Groups in the Cecum Digesta

The gut microbiota in the cecum was analyzed by real-time PCR according to previous studies [30,31]. DNA of cecum digesta was extracted using QIAamp^®^ Fast DNA Stool Mini kit (Qiagen) according to the manufacturer’s protocol. DNA was mixed with PowerUp SYBR Green Master Mix (Thermo Fisher Scientific, Waltham, MA, USA) and oligonucleotide primers for predominant bacterial groups (Table S2). Amplification of the DNA was performed using an Applied Biosystems Quant3 Real-Time PCR System (Thermo Fisher Scientific). From the obtained threshold cycle (Ct) values, colony-forming units (CFU) in the cecum were determined from a calibration curve prepared using Ct values after serially diluting a DNA solution extracted from each standard bacterial strain (Table S2).

### 2.6. Expression Analysis of mRNA Related to Immunity in the Ileum

Total RNAs in the ileum were extracted using an RNeasy Mini Kit (Qiagen). mRNA expression related to intestinal immunity and cytokines was analyzed by real-time PCR using an Applied Biosystems Quant3 Real-Time PCR System and PowerUp SYBR Green Master Mix (Thermo Fisher Scientific) with cDNA synthesized from RNA. Primer sequences are given in Table S3. The 2^−ΔΔCT^ method was used for mRNA expression analysis. We used 36B4 as a reference gene and calculated ΔCT compared to the 36B4. Next, we calculated ΔΔCT as the difference between ΔCT for the C group and HGB group in terms of cDNA solution added to each primer. Relative expression levels are presented as fold changes to the C group (arbitrary units).

### 2.7. Statistical Analysis

All statistical analyses were performed using R Studio (ver. 1.3.1093, R-Tools Technology Inc., Richmond Hill, ON, Canada). Data are presented as mean ± standard error (SE) of the mean. For each parameter, Student’s *t*-test was used when the data were based on a normal distribution. If a normal distribution was not confirmed, the Wilcoxon test was used. Significant differences were appraised using a two-side test with an α level of 0.05. The relationships between the sIgA and parameters related to intestinal immunity were analyzed by Spearman’s rank correlation coefficient.

## 3. Results

### 3.1. KEGG Enrichment Analyses of DEGs by Using DNA Microarray Data

We performed KEGG enrichment analysis of DEGs using DNA microarray data of the ileum of mice fed a high-fat diet containing barley flour. The expression levels of 3065 genes were determined as DEGs. The DEGs up-regulated in the barley group showed an enrichment in pathways related to intestinal immunity, such as “Cytokine–cytokine receptor interaction (mmu04060)” and “B cell receptor signaling pathway (mmu04662)” (Table S4, Figure S2). By contrast, only one pathway (mmu04740: Olfactory transduction) was identified from DEGs down-regulated in the barley group (data not shown).

### 3.2. Food Intake, Body Weight, and Organ Weight

In the animal study, intake of the experimental diet showed no significant differences with the control group. Therefore, mice in both groups were determined to have been fed a similar level of energy during the study period (Table S5). However, body weight gain and final weight in the HGB group were significantly lower than the control group (*p* < 0.05). As a result, food efficiency ratio in the HGB group was lower than the C group (*p* < 0.05). The organ weight of liver, retroperitoneal, and mesenteric fats were lower in the HGB group than the C group (*p* < 0.05), while the cecum digesta was significantly higher (*p* < 0.05).

### 3.3. Secretory Immunoglobulin A (sIgA) Concentration in the Cecum and Serum

**Figure 1:**
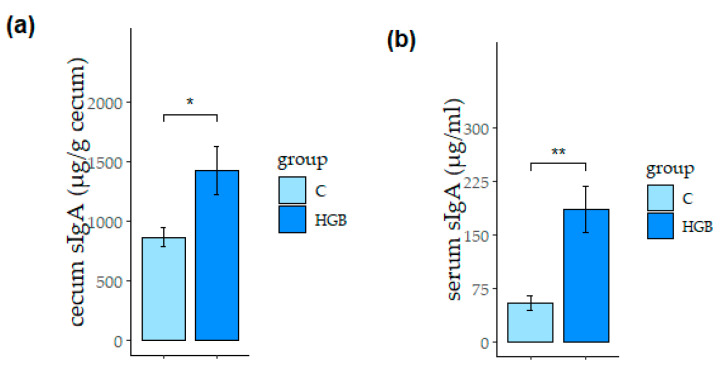
The concentration of sIgA in the cecum (**a**) and serum (**b**). Values are means ± SE, *n* = 8. * *p* < 0.05, ** *p* < 0.01 indicates a significant difference between each group, C: control group, HGB: high β-glucan barley group. sIgA, secretory immunoglobulin A.

The concentrations of sIgA in the cecum and serum are shown in Figure 1. Cecum (Figure 1a) and serum (Figure 1b) sIgA levels in the HGB group were significantly higher than in the C group (*p* < 0.05). The concentrations of serum IL-10 and IL-6 were not statistically different between the two groups (Figure S3).

### 3.4. SCFA and Organic Acid Concentration in the Cecum Digesta and Feces

**Figure 2:**
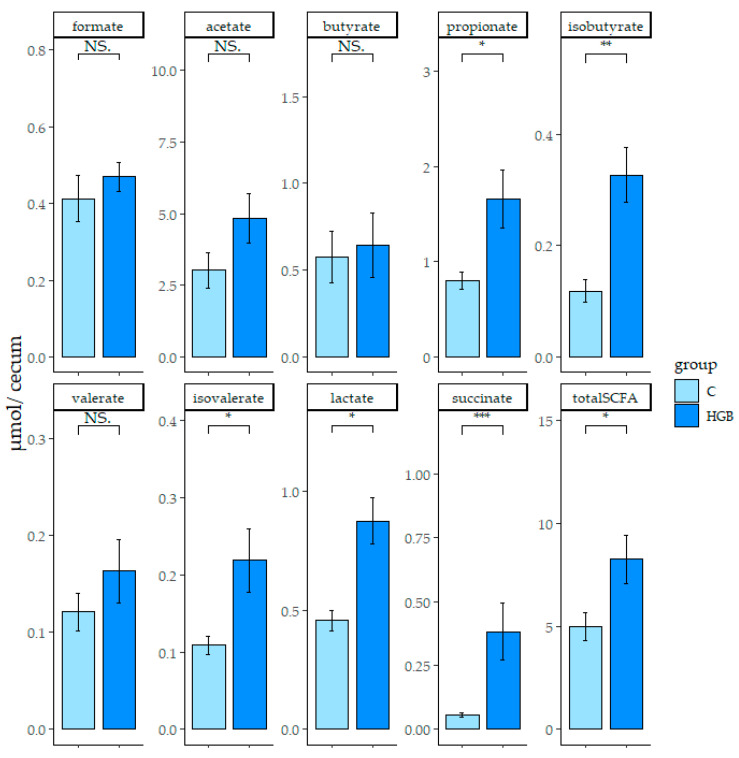
The concentration of short-chain fatty acids (SCFAs), lactate, and succinate in the cecum digesta. Values are means ± SE, *n* = 8. * *p* < 0.05, ** *p* < 0.01, *** *p* < 0.001 showed a significant difference between each group, “NS” is not significant. C: control group, HGB: high β-glucan barley group.

The concentration of SCFAs and organic acids in the cecum digesta are shown in Figure 2. The total level of SCFAs, propionate, isobutyrate, isovalerate, lactate, and succinate concentrations in the HGB group were significantly higher than the C group (*p* < 0.05). The level of acetate also displayed a slight increase in the HGB group compared with the C group, although this was not statistically significant. The concentration of butyrate, propionate, isobutyrate, valerate, isovalerate, lactate, and succinate in the feces were significantly higher in the HGB group than the C group (*p* < 0.05) (Figure S4).

### 3.5. Counts of Predominant Bacterial Groups in the Cecum Digesta

Bacterial counts of microbiota at the phylum and genus levels in the cecum digesta are shown in Table 1. At the phylum level, the bacterial count of *Bacteroidetes*, *Firmicutes*, and total bacteria were significantly higher in the HGB group than the C group (*p* < 0.05). At the genus level, the bacterial counts of the *Bacteroides*, *Bifidobacterium*, *Lactobacillus,* and *Atopobium* cluster were significantly higher in the HGB group than the C group (*p* < 0.05).

### 3.6. Expression of mRNA Related to the Immune System in the Ileum

**Figure 3:**
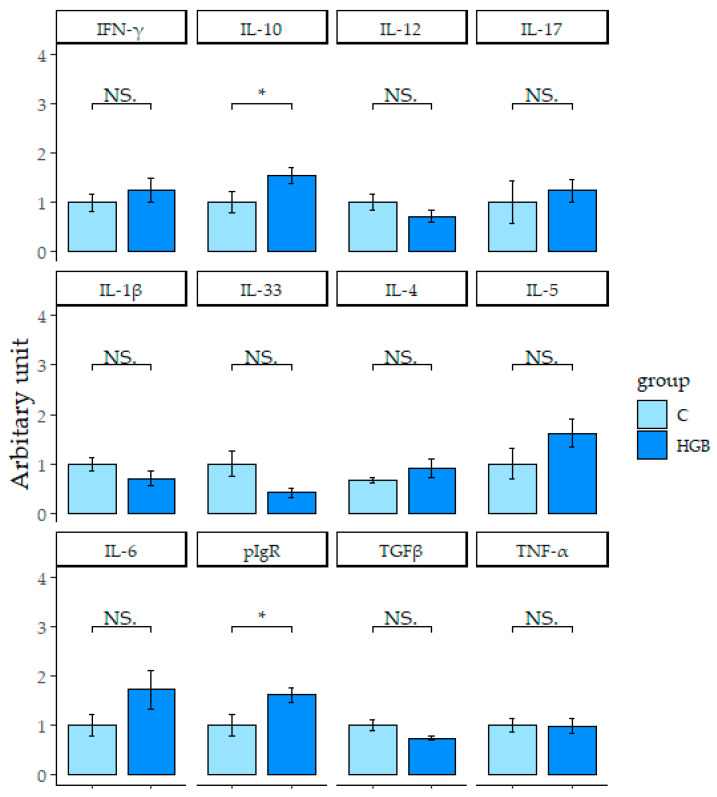
The expression levels of mRNA in the ileum. Values are means ± SE, *n* = 8. * *p* < 0.05 indicates a significant difference between each group, “NS” is not significant. C: control group, HGB: high β-glucan barley group. *IFN-γ*, interferon gamma; *IL-10*, interleukin 10; *IL-12*, interleukin 12; *IL-17*, interleukin 17; *IL-1β*, interleukin 1 beta; *IL-33*, interleukin 33; *IL-4*, interleukin 4; *IL-5*, interleukin 5; *IL-6*, interleukin 6; *pIgR*, polymeric immunoglobulin receptor; *TGFβ*, transforming growth factor beta; *TNF-α*, tumor necrosis factor-α.

Ileum mRNA expression levels related to the immune system are shown in Figure 3. In the HGB group, mRNA expression levels of *IL-10* were significantly higher than the C group (*p* < 0.05). mRNA expression levels of other cytokines (*IFN-γ*, *IL-12*, *IL-1β*, *IL-4*, *IL-5*, *IL-6*, *IL-33*, transforming growth factor beta (*TGF-β*), tumor necrosis factor-α (*TNF-α*)*,* and *IL-17* were not statistically different between the two groups. mRNA expression level of the polymeric immunoglobulin receptor (*pIgR*) in the HGB group was significantly higher than the C group (*p* < 0.05).

### 3.7. Correlation Analysis between sIgA Concentration and Parameters Related to the Immune System in the Ileum and Cecum

**Figure 4:**
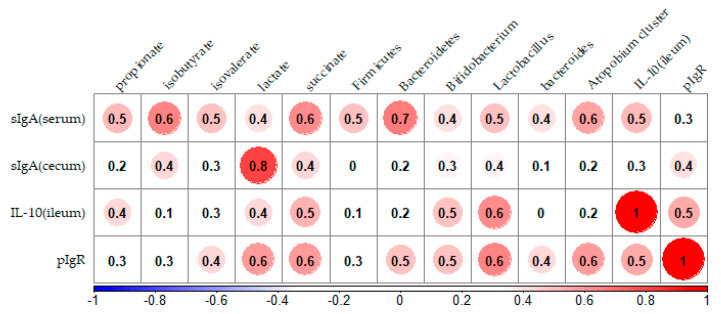
Result of the Spearman’s rank correlation coefficients analysis between the concentration of serum and cecum sIgA and parameters related to intestinal immunity. The values in the figure show the correlation, and red circles highlight a significantly positive correlation. sIgA, secretory immunoglobulin A; *IL-10*, interleukin 10; *pIgR*, polymeric immunoglobulin receptor.

The correlation coefficients between the concentration of sIgA and parameters related to immunity response are shown in Figure 4. Parameters that were significantly different in the HGB group compared to the C group were used in the analysis. A positive correlation was identified between sIgA in the cecum and isobutyrate, lactate, succinate, *Lactobacillus*, and *pIgR* (*p* < 0.05). A strong positive correlation was identified between sIgA in the serum and most gut bacteria (*p* < 0.05).

## 4. Discussion

In this study, we investigated the effect of HGB flour on the immune system in diet-induced obese mice. Intake of HGB flour with a high-fat diet increased the concentration of sIgA in the cecum and serum. A positive correlation was confirmed between the intestinal flora and intestinal fermentation metabolites, which are related to an increase in the level of IgA. In addition, the increase in mRNA expression of *IL-10* by HGB intake suggests that SCFAs and lactic acid may regulate the Tregs response and affect the immune response.

First, we used previously determined DNA microarray data of the ileum [28] to establish how HGB flour intake affects gene expression related to the immune system in the high-fat diets. KEGG enrichment analysis of DEGs suggested that HGB flour altered gene expression involved in several metabolic pathways of the ileum. Specifically, the B cell receptor signaling pathway (mmu04662) was up-regulated in mice fed the HGB diet. The B cell receptor is connected with the function and regulation of B cells differentiated into IgA plasma cells. The IgA antigen contributes an important role in the immune response in mucous membranes, such as the digestive tract, and is the most abundant antigen on the mucosal surface [32]. In a recent study, IgA was shown to mediate the modulation of microbiota in the digestive tract [33]. A previous study revealed that the microbiota composition differed significantly between immunodeficient mice and wild-type mice [34]. Thus, our findings suggest that barley affects the immune system, such as IgA secretion, via regulation of gut microbiota.

Next, we investigated changes to the immune system and microbiota using high-fat model mice. A previous study indicated that C57BL/6J mice fed a high-fat diet (60 kcal%fat) for 14 weeks had reduced IgA-producing cells in the gut-associated immune system and sIgA in the colon compared to mice fed a normal diet (16 kcal%fat) [35]. Nonetheless, we found an increase in the concentration of sIgA in the cecum and serum of the HGB group compared to the C group. Additionally, these results were also supported by the increase in the mRNA expression of *pIgR*, which binds to IgA and transports sIgA to the intestinal lumen side. Moreover, a positive correlation was found between the concentration of sIgA in the serum or cecum with the increased number of gut microbiota in mice from the HGB group. Thus, this observation may be the result of a prebiotic effect. Several studies have reported the effects of cereal dietary fiber on gut microbiota and the immune response. Yuri et al. showed that C57BL/6J mice fed oat-derived β-glucan formulations had higher levels of total serum immunoglobulins, which increased their resistance to the murine pathogen *Eimeria vermiformis* [36]. Another study indicated that a long-term intake of wheat bran significantly increased fecal sIgA and IgA bacteria in male BALB/c mice via modulation of the gut microbiota [37]. These reports back up our present findings. Moreover, our results show that ingestion of HGB flour up-regulates immune function, especially the secretion of IgA under a high-fat diet.

The concentration of total SCFAs and propionate in the cecum digesta were significantly higher in the HBG group compared to the C group. Smith et al. reported that acetate and propionate activated the migratory properties of Tregs through mediated G protein-coupled receptor 43 (*GPR43*) [38]. Several studies have addressed how Tregs regulate the IgA secretion of germinal center B cells [39,40]. Moreover, IL-10 secreted by Tregs suppresses the production of inflammatory cytokines by acting on macrophages and dendritic cells. Our results showed that HGB flour increased mRNA expression of *IL-10* in the ileum, and a positive correlation was confirmed between propionate and *IL-10*. Thus, intake of barley flour may affect the migration of Tregs and secretion of IL-10 via propionate. Indeed, a previous study showed that gut microbiota-derived SCFAs promote IL-10 production [41]. However, no significant difference was found in other inflammatory cytokines in the ileum and IL-6 and IL-10 level in serum. Although other studies reported that a long-term intake of oat β-glucan lowered the levels of inflammatory cytokines in the colon [42], we found no convincing evidence of an anti-inflammatory effect. Further studies are needed to investigate the anti-inflammatory effect of HGB flour.

A previous study indicated that butyrate strongly induces the differentiation of T regs in the colon through activation of the forkhead box protein P3 (*Foxp3*) gene [43]. Foxp3 plays an essential role in Tregs differentiation, functional expression, and maintenance of differentiation state [44]. However, our results showed no significant difference between the experimental diets in terms of the concentration of butyrate or butyrate-producing bacteria, such as *Clostridium leptum group* and the *Clostridium coccoides subgroup*. Moreover, there was no significant difference in the gene expression level of *Foxp3* (data not shown). Therefore, we concluded that the observed increase in sIgA is due to HGB intake promoting Tregs migration rather than the differentiation of Tregs.

In this study, a positive correlation was observed between the concentration of sIgA in the serum or cecum and *Lactobacillus* or lactate. Moreover, the bacterial count of the *Atopobium* cluster was significantly higher in the HGB group. Since the *Atopobium* cluster produces lactate as major metabolite, we proposed that these bacteria contributed to the observed increase in the concentration of lactate [45]. There are numerous reports on the antigenic effect of some *Lactobacillus* species on the gut immune system [46,47]. In particular, in vitro studies have shown that *Lactobacillus reuteri* and *Lactobacillus casei* increase the expression of *IL-10* [48]. Therefore, the correlation coefficient observed in this study suggests that increased levels of lactic acid-producing bacteria brought about by an intake of HGB flour may have enhanced the concentration of sIgA through the intestinal immune response. Our previous investigations have shown that the ingestion of HGB flour increases the levels of lactic acid-producing bacteria [49], but this is the first such study to report an effect on immune function. Further research is needed to clarify the species of lactic acid bacteria increased by the intake of HGB flour and to investigate their functions.

The β-(1-3), (1-6) glucan derived from yeast and mushrooms bind to the dectin-1 and Toll-like receptors present in immune cells, such as monocytes and dendritic cells, and influence the adaptive immune response, such as the production of cytokines and chemokines [50,51]. These effects had been attributed to the β-(1-3) linkage and structure of the β-(1-6) branching at a certain site that enhances interaction with specific receptors [52]. Results from a previous in vitro study suggested that barley-derived β-glucan may, at least in part, also affect the immune system via dectin-1 mediated changes [53]. However, it was shown that the affinity for barley β-glucan was weaker than for the continuous β-(1-3) glucan (sizofiran) [54]. Furthermore, because β-(1-3), (1-4) glucans of cereals do not contain β-(1-6) branches, β-glucans of cereals and yeast/mushrooms differ considerably in terms of solubility, molecular weight, and branching structure. Therefore, we propose that the increased level of sIgA observed in the HGB group was influenced by SCFAs and gut microbiota rather than dectin-1 signaling. Further studies are required to clarify the affinity and function for dectin-1 binding of β-(1-3), (1-4) glucan.

## 5. Conclusions

Our results indicated that an intake of HGB flour increases the concentration of sIgA in the serum and cecum under high-fat diet conditions. These findings suggest that this effect is mediated by an alternation in the gut microbiota and a subsequent increase in the levels of organic acids, including SCFAs, generated by intestinal fermentation of barley β-glucan. Since the mRNA expression level of *IL-10* was elevated in the ileum, it may be affected by Tregs, which are IL-10-producing cells. These findings will help to explain how the prebiotic effects of barley flour can improve the immune system or alleviate a weakened immune system deteriorated by a poor diet.

## Figures and Tables

**Table 1:** Counts of the predominant bacterial groups in the cecum digesta between the control and HGB groups.

LogCFU/g (Cecum Digesta)	Control	HGB
Phylum		
Total bacteria	13.3 ± 0.3	14.9 ± 0.4 *
*Bacteroidetes*	10.0 ± 0.2	11.2 ± 0.2 *
*Firmicutes*	12.7 ± 0.1	12.9 ± 0.1 *
*Actinobacteria*	9.6 ± 0.4	10.1 ± 0.1
Genus		
*Bacteroides fragilis group*	9.7 ± 0.1	10.3 ± 0.2 *
*Bifidobacterium*	10.3 ± 0.3	11.1 ± 0.1 *
*Lactobacillus*	10.2 ± 0.2	11.3 ± 0.1 *
*Prevotella*	7.4 ± 0.1	7.6 ± 0.1
*Clostridium coccoides group*	9.8 ± 0.2	10.3 ± 0.1
*Clostridium leptum subgroup*	11.2 ± 0.2	11.7 ± 0.1
*Atopobium* cluster	7.8 ± 0.2	9.1 ± 0.2 *

* *p* < 0.05 significantly different between the control and HGB group. Values are means ± SE (*n* = 8). HGB: High β-glucan barley group.

## Supplementary Materials

The following are available online at https://www.mdpi.com/2072-6643/13/3/907/s1, Table S1: composition of the control and HGB diets, Table S2: List of standard bacterial strain and primer sequence for real-time PCR, Table S3: List of primers used for real-time PCR, Table S4: KEGG enrichment analysis with up-regulated DEGs in the ileum of barley group by using DAVID database, Table S5: Final weight, body weight gain, food intake, and organ weight in mice fed the control and HGB diets, Figure S1: Flow chart showing the study design of the animal studies, Figure S2: DEGs up-regulated by barley group involved in B cell receptor signaling pathway (extracted from Kyoto Encyclopedia of Genes and Genomes pathway), Figure S3: The concentrations of *IL-10* and *IL-6* in serum, Figure S4: The concentrations of SCFAs and organic acids in feces.
