# Supplementary material for: Ingestion of High β-Glucan Barley Flour Enhances the Intestinal Immune System of Diet-Induced Obese Mice by Prebiotic Effects
Source: Nutrients. 2021 Mar 11;13(3):907. doi: 10.3390/nu13030907 (PMC7999470; doi:10.3390/nu13030907)
Supplement: Supplementary file 1 [file nutrients-13-00907-s001.pdf]

Table S1. Composition of the control and HGB diets.

(g/kg diet)		
	**Control**	HGB
Casein	200	157.4
Corn starch	197.5	-
α-Corn starch	132	27.3
Sucrose	100	100
Soybean oil	70	70
Lard	200	192.4
Cellulose	50.0	-
Barley flour (“White Fiber”)	-	402.4
AIN-93G mineral mixture	35	35
AIN-93 vitamin mixture	10	10
L-cystine	3	3
Choline bitartrate	2.5	2.5
t-butylhydroquinone	0.014	0.014

Barley flour (“White Fiber”) from the Hakubaku Co. Ltd (Yamanashi, Japan), White fiber is contained β-glucan, 8.0%; total dietary fiber, 12.4%; protein, 8.4%; fat, 1.9%.
HGB: High β-glucan barley group.

Table S2. List of standard bacterial strain and primer sequence for real time PCR.

Phylum	Forward (Fw) / Reverse (Rv)	Primer sequence	Standard bacteria strain.	Strain No.	Reference
Bacteroidetes	Fw	5'-CRAACAGGATTAGATACCCT-3'	*Bacteroides fragilis*	JCM11019	De Gregoris *et al*: J. Microbial Methods, Sep;86(3): 351-6, 2011.
	Rv	5'-GGTAAGGTTCCTCGCGTAT-3'			
Firmicutes	Fw	5'-TGAAACTYAAAGGAATTGACG-3'	*Ruminococcus albus*	JCM14654	De Gregoris *et al*: J. Microbial Methods, Sep;86(3): 351-6, 2011.
	Rv	5'-ACCATGCACCACCTGTC-3'			
Actinobacteria	Fw	5′-TACGGCCGCAAGGCTA-3′	*Bifidobacterium longum*	JCM1217	De Gregoris *et al*: J. Microbial Methods, Sep;86(3): 351-6, 2011.
	Rv	5′-TCRTCCCCACCTTCCTCCG-3′			
Total bacteria	Fw	5′-AAACTCAAAKGAATTGACGG	*Ruminococcus albus*	JCM14654	De Gregoris *et al*: J. Microbial Methods, Sep;86(3): 351-6, 2011.
	Rv	5′-CTCACRRCACGAGCTGAC-3′			
**Genus**	**Fw / Rv**	**Primer sequence**	**Standard bacteria**	**Strain No.**	**Reference**
Bacteroides fragilis group	Fw	5'-GTCAGTTGTGAAAGTTTGC-3'	*Bacteroides fragilis*	JCM11019	Bernard AE *et al*: Appl Environ Microbiol, 66: 1587-94, 2000.
	Rv	5'-CAATCGGAGTTCTTCGTG-3'			
Lactobacillus	Fw	5'-TGGAAACAGGTGCTAATAGGC-3'	*Lactobacillus ramnosus*	ATCC8530	Roy Byun *et al*: J Clin Microbiol, 42(7): 3128-36, 2004.
	Rv	5'-GTCCATTGTGGAAGATTCCC-3'			
Bifidobacterium	Fw	5'-AGGGTTCGATTCTGGCACAG-3'	*Bifidobacterium longum*	JCM1217	Kok RG *et al*: Appl Environ Microbiol, 62: 3668-72, 1996.
	Rv	5'-CATCCGGCATTACCACCC-3'			
Prevotella	Fw	5'-CACCAAGGCGACGATGA-3'	*Prevotella malaninogenica*	JCM6325	Larsen *et al*: PLos ONE, 5: e9085, 2010.
	Rv	5'-GGATAACGCCTGGACCT-3'			
Clostridium leptum subgroup	Fw	5'-GCACAAGCAGTGGAGT-3'	*Ruminococcus albus*	JCM14654	Matsuki *et al*: Appl Environ Microbiol, 70: 7220-8, 2004.
	Rv	5'-CTTCCTCCGTTTTGTCAA-3'			
Clostridium coccoides group	Fw	5'-AAATGACGGTACCTGACTAA-3'	*Clostridium coccoides*	JCM1395T	Matsuki *et al*: Appl Environ Microbiol, 70: 7220-8, 2004.

	Rv	5'-CTTTGAGTTTCATTCTTGCGAA-3'			
Atopobium cluster	Fw	5'-GGGTTGAGAGACCGACC-3'	*Collinsella aerofacience*	JCM10188	Matsuki *et al*: Appl Environ Microbiol, 70: 7220-8, 2004.
	Rv	5'-CGGRGCTTCTTCTGCAGG-3'			

R =A or G, K=T or G, Y=C or T.

Table S3. List of primers used for real-time PCR.

gene symbol	Forward	Reverse	reference
IFN-γ	5'-GCATCTTGGCTTTGCAGCT-3'	5'-CCTTTTTCGCCTTGCTGTTG-3'	Takiguchi, H *et al*: J Oral Sci, 54: 23-32, 2012.
IL-1β	5'-CGCAGCAGCACATCAACAAGAGG-3'	5'-TGTCCTCATCCTGGAAGGTCCAC-3'	Aoe, S *et al*: Int J Hum Cult Stud, 26: 277-282, 2016.
IL-4	5'-CTTCCAAGGTGCTTCGCATA-3'	5'-AAGCCCGAAAGAGTCTCTGC-3'	Yoon *et al*: BMC Complement Altern Med, 15: 353, 2015.
IL-5	5'-CCCCCACGGACAGTTTGATT-3'	5'-TGAGGCTTCCTGTCCCTACT-3'	Primer design was performed by NCBI Primer-Blast tool.
IL-6	5'-CGGCCTTCCCTACTTCACAA-3'	5'-TCTGCAAGTGCATCATCGTT-3'	Mio, K *et al*: nutrients, 12: 3546, 2020.
IL-10	'5'-TGGACAACATACTGCTAACC-3'	5'-GGATCATTTCCGATAAGGCT-3'	Paiva *et al*: BMC Microbiology, 13: 69, 2013.
IL-12	5'- AGCACCAGCTTCTTCATCAGG -3'	5'-GCGCTGGATTCGAACAAAG-3'	Paiva *et al*: BMC Microbiology, 13: 69, 2013.
IL-17	5'-GCTCCAGAAGGCCCTCAGA-3'	5'-CTTTCCCTCCGCATTGACA-3'	Paiva *et al*: BMC Microbiology, 13: 69, 2013.
IL-33	5'-GGGCTCACTGCAGGAAAGTA-3'	5'-TTTGCCGGGAAATCTTGGA-3'	Primer design was performed by NCBI Primer-Blast tool.
TNF-α	5'-ACCCTCACACACTCAGATCATCTTC-3'	5'-TGGTGGTTTGCTACGACGT-3'	Aoe, S *et al*: Int J Hum Cult Stud, 26: 277-282, 2016.
TGFβ	5'-AGGGCTACCATGCCAACTTC-3'	5'-CCACGTAGTAGACGATGGGC-3'	Lai D *et al*: PLoS One, 9(5): e98749, 2014.
pIgR	5'-TTGTTCACGCTCTTGGTAACTG-3'	5'-ACAGGCCTCGGTTACTGGTACC-3'	Blanch, VJ *et al*: J Immunol, 62: 1232-5, 1999.
Reference			
36B4	5'-GGCCCTGCACTCTCGCTTTC-3'	5'-TGCCAGGACGCGCTTGT-3'	Aoe, S *et al*: nutrients, 11: 1674, 2019.

R =A or G. IFN-γ, interferon gamma; IL-10, interleukin 10; IL-12, interleukin 12; IL-17, interleukin 17; IL-1β, interleukin 1 beta; IL-33, interleukin 33; IL-4, interleukin 4; IL-5, interleukin 5; IL-6, interleukin 6; pIgR, polymeric immunoglobulin receptor; TGFβ, transforming growth factor beta; TNF-α, tumor necrosis factor-α.

Table S4. KEGG enrichment analysis with upregulated DEGs in the ileum of barley group by using DAVID database.

ID	Pathway name	Count	P value	FDR
mmu00591	Linoleic acid metabolism	12	8.3e-6	2.0e-3
mmu00590	Arachidonic acid metabolism	15	3.0e-5	3.6e-3
mmu04640	Hematopoietic cell lineage	14	7.8e-5	6.2e-3
mmu00830	Retinol metabolism	14	1.3e-4	7.4e-3
mmu04060	Cytokine-cytokine receptor interaction	25	1.5e-4	7.4e-3
mmu05150	Staphylococcus aureus infection	10	2.9e-4	1.1e-2
mmu05340	Primary immunodeficiency	8	5.8e-4	2.0e-2
mmu05204	Chemical carcinogenesis	13	6.7e-4	2.0e-2
mmu04514	Cell adhesion molecules (CAMs)	18	7.5e-4	2.0e-2
mmu04662	B cell receptor signaling pathway	11	9.1e-4	2.2e-2
mmu00140	Steroid hormone biosynthesis	12	1.4e-3	3.1e-2
mmu04610	Complement and coagulation cascades	11	1.7e-3	3.4e-2

FDR were adjusted by using Benjamini–Hochberg algorithm.

Table S5. Final weight, body weight gain, food intake, and organ weight in mice fed the control and HGB diets.

	**Control**	**HGB**
Initial weight (g)	20.0 ± 0.3	20.0 ± 0.4
Final weight (g)	42..4 ± 1.5	38.4 ± 1.2*
Body weight gain (g/day)	0.25 ± 0.02	0.20 ± 0.01*
Food intake (g/day)	2.85 ± 0.06	2.82 ± 0.04
Food efficiency ratio (%)	8.7 ± 0.4	7.2 ± 0.3*
Liver (g)	1.5 ± 0.1	1.2 ± 0.1*
Cecum with digesta (g)	0.2 ± 0.02	0.4 ± 0.03*
Epididymal fat (g)	2.3 ± 0.1	2.2 ± 0.2
Retroperitoneal fat (g)	1.0 ± 0.1	0.7 ± 0.1*
Mesenteric fat (g)	1.1 ± 0.2	0.6 ± 0.1*

Values are mean ± SE (n=8).* p<0.05 showed significantly different among each group. Food efficiency ratio (%) = Body weight gain/Food intake × 100. HGB: High β-glucan barley group.

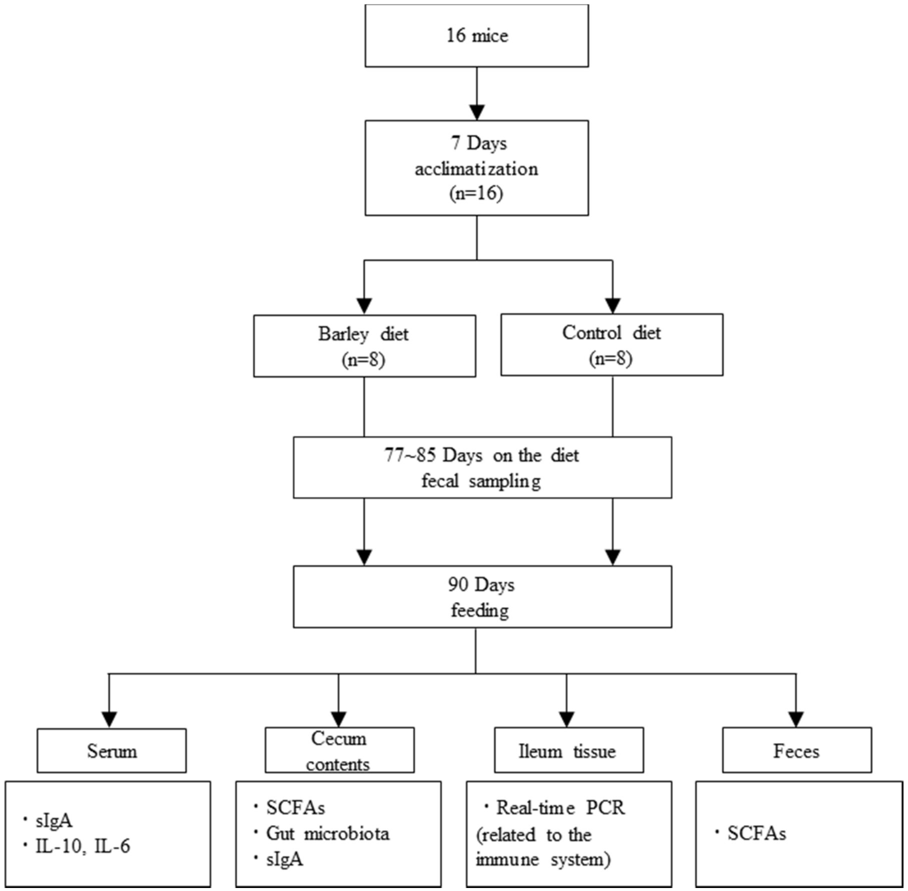

Figure S1. Flow chart showing the study design of the animal studies. sIgA, secretory immunoglobulin A; SCFAs, Short-chain fatty acids.

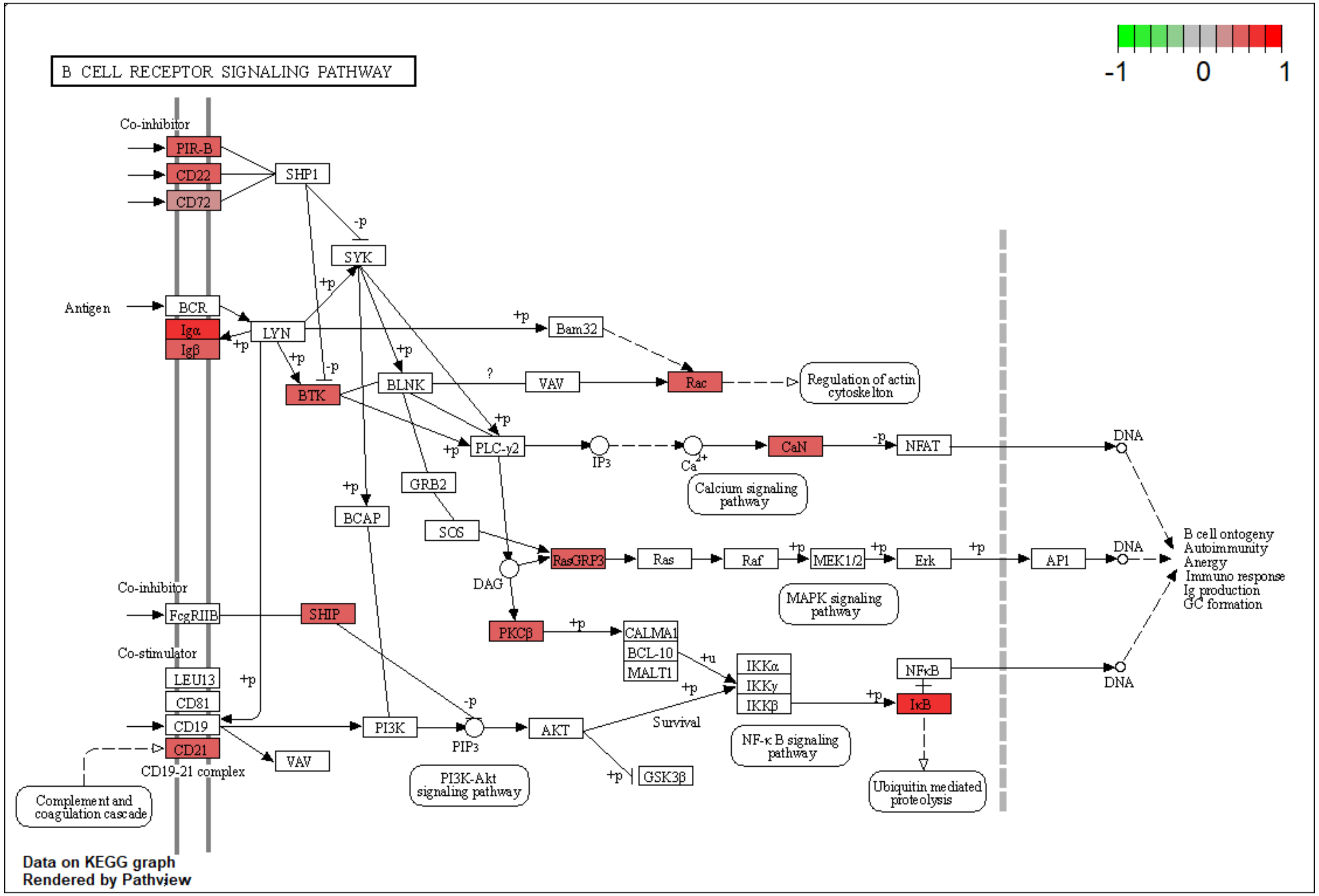

Figure S2. DEGs up-regulated by barley group involved in B cell receptor signaling pathway (extracted from Kyoto Encyclopedia of Genes and Genomes pathway). DEGs: Differential expressed genes had selected the cut-off criteria (Log-ratio > 1.3 fold in the barley group compared with the control group). The color depth in the figure indicates the Log-ratio values.

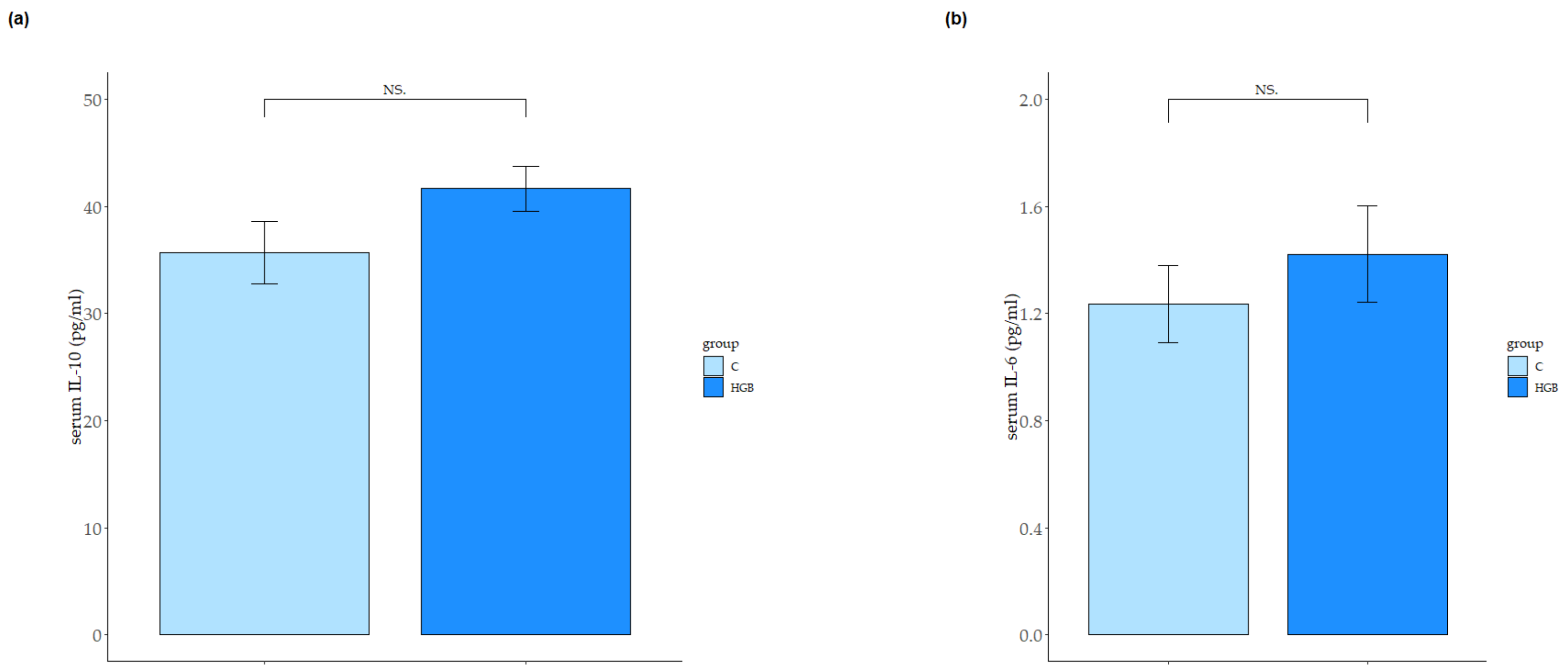

Figure S3. The concentration of IL-10 (a) and IL-6 (b) in serum. Values are means ± SE, n=8. C: control group, HGB: high β-glucan barley group. "NS" is not significant difference between each group.

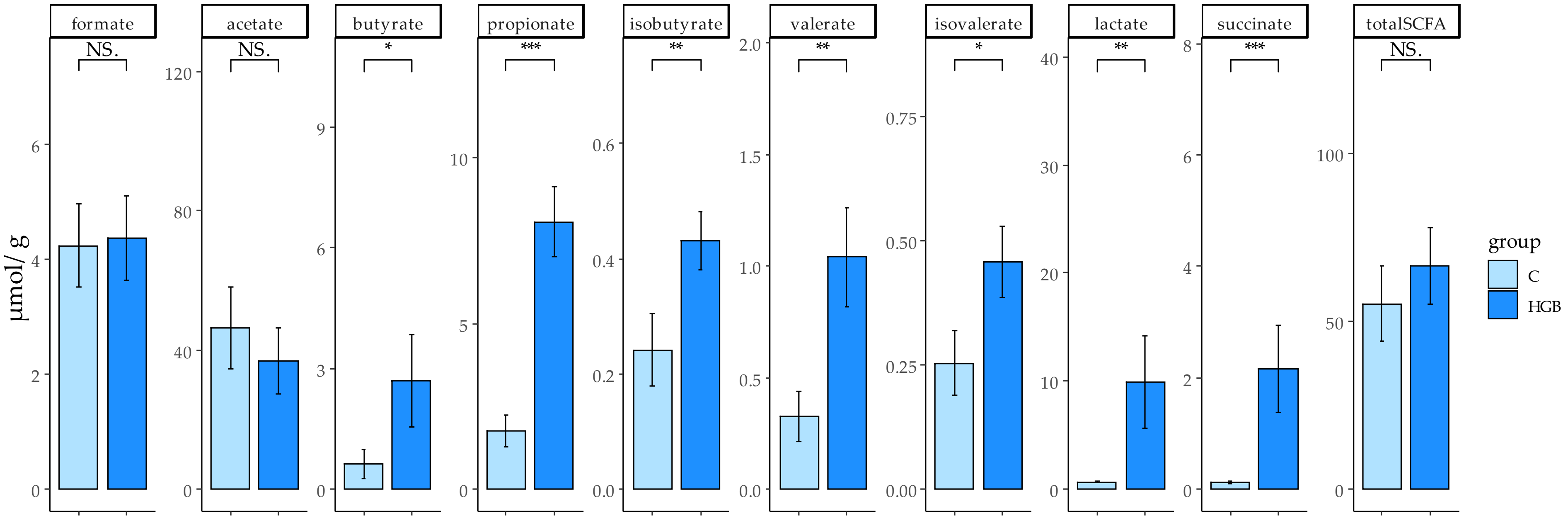

Figure S4. The concentration of SCFAs and organic acids in feces. Values are means ± SE, n=8. * p<0.05, ** p<0.01, *** p<0.001 showed a significant difference between each group, "NS" is not significant. C: control group, HGB: high β-glucan barley group. C: control group, HGB: high β-glucan barley group.
